# Circulating miRNA-486 as a novel diagnostic biomarker for right ventricular remodeling

**DOI:** 10.3389/fcvm.2025.1518022

**Published:** 2025-01-29

**Authors:** Huiling Cai, Cheng Yu, Xiuchuan Li, Xuenan Wang, Yongjian Yang, Cong Lan

**Affiliations:** ^1^ Department of Cardiology, School of Clinical Medicine, Southwest Medical University, Luzhou, Sichuan, China; ^2^ Department of Cardiology, The General Hospital of Western Theater Command, Chengdu, Sichuan, China; ^3^ Department of Cardiology, Fujian Heart Center, Provincial Institute of Coronary Disease, Fujian Medical University Union Hospital, Fuzhou, Fujian, China; ^4^ College of Medicine, Southwest Jiaotong University, Chengdu, Sichuan, China

**Keywords:** miRNA-486, diagnostic biomarker, pulmonary hypertension, right ventricular remodeling, pulmonary artery banding

## Abstract

**Objective:**

Clinical practice currently faces a significant shortfall in specific biomarkers needed for diagnosing right ventricular (RV) remodeling in patients with pulmonary hypertension (PH). While small noncoding microRNAs (miRNAs) are crucial regulators of RV remodeling, the biomarker potential of serum miRNAs in this process is little known. This study systematically screened and identified candidate serum miRNAs as potential diagnostic biomarkers for RV remodeling in PH patients.

**Methods:**

Pulmonary artery banding (PAB) was performed in Sprague-Dawley (SD) rats and RV modeling was measured by echocardiographic and histological analyses 4 weeks after surgery. High-throughput miRNA sequencing of serum samples was performed to profile differentially-expressed miRNAs (dif-miRNAs) and preliminarily screen candidate miRNAs. The diagnostic power of the candidate miRNA was further validated in 100 patients [20 with adaptive RV pressure overload; 20 with maladaptive RV pressure overload; 20 with left heart failure (LHF); 19 with left ventricular hypertrophy and 21 controls].

**Results:**

PAB rats exhibited severe RV hypertrophy, fibrosis and enlargement of RV cardiomyocytes compared with sham group. MiRNA sequencing analyses revealed 19 dif-miRNAs (12 upregulated and 7 downregulated) between the two groups. Among the 12 upregulated miRNAs, miRNA-486 exhibited highest elevation in PAB group and was supposed to be the candidate biomarker for RV modeling. Serum miRNA-486 levels were lower in control and left ventricular hypertrophy (LVH) patients compared to PH patients, and significantly higher in maladapted RV patients than in adapted RV patients. Serum miRNA-486 was significantly higher in LHF patients compared to controls, but still significantly lower than in PH patients. In receiver operating characteristic (ROC) analysis, serum miRNA-486 was a good predictor of RV maladaptation in PH patients (cut-off value 3.441, AUC 0.8625), which was not significantly different from B-type natriuretic peptide (BNP). Elevated serum miRNA-486 levels (≥3.441) were associated with reduced TAPSE/PASP ratios and increased BNP levels.

**Conclusions:**

Serum miRNA-486 has the potential to be a valuable noninvasive biomarker for diagnosing RV remodeling in patients with PH.

## Introduction

RV failure frequently occurs in patients suffering from pulmonary hypertension (PH). Substantial evidence indicates that RV dysfunction is a hallmark of poor clinical outcome for PH patients, which is independently correlated with reduced survival and overall prognosis (1–3). Furthermore, progressive RV remodeling characterized by structural and functional changes leads to a series of clinical complications that exacerbate the severity of PH, ultimately leading to increased hospitalization and mortality. While current clinical treatments for PH, focusing on the endothelin, prostacyclin pathways, and nitric oxide to balance vasodilation and vasoconstriction, have improved short-term prognosis, long-term outcomes remain poor due to maladaptive RV remodeling and RV failure (4–6). Accurate predictive diagnosis is essential for preventing RV failure and optimizing PH treatment. Brain natriuretic peptide (BNP) and N-terminal brain natriuretic peptide precursor (NT-proBNP) are widely used by clinicians to assess the presence and severity of heart failure, but their specificity is low and elevated BNP levels can also result from conditions like left or right heart failure, total heart failure, pneumonia, myocardial ischemia, and renal failure (7, 8). Thus, more researches are needed to identify novel noninvasive biomarkers with predictive value in diagnosing pathological RV remodeling and dysfunction.

miRNAs constitute a distinct class of endogenous, single-stranded non-coding RNAs, ranging from approximately 21–25 nucleotides in length. These molecules predominantly function to modulate gene expression by modulating the stability and translation of target messenger RNAs (mRNAs) at the post-transcriptional level (9). MiRNAs are crucial regulators of cardiovascular development, influencing processes like angiogenesis, cardiomyocyte differentiation, cell cycle, cardiac electrical activity, and cardiomyocyte growth (10). Meanwhile, the essential of miRNAs in cardiovascular diseases have been intensively studied, such as atherosclerosis and arrhythmias (11–13). Furthermore, circulating miRNAs are cell-or tissue-specific, relatively stable in serum, and resistant to degradation factors (14). These properties make serum miRNAs possible promising biomarkers for predictive diagnosis of cardiovascular disease (CVD) (15, 16). However, although the expression, regulation, and function of miRNAs in RV remodeling have been intensively studied, the information on the diagnostic potential of circulating miRNAs for RV remodeling remains limited.

The objective of this study was to: (1) Establish a comprehensive model of RV remodeling in rats by PAB and revealing circulating miRNA profiles to screen potential biomarkers via high throughout miRNA sequencing for serum samples; (2) validate the diagnostic efficacy of the candidate biomarker in patients with adaptive RV pressure overload, maladaptive RV pressure overload, left ventricular hypertrophy, left heart failure, and healthy controls.

## Methods

### PAB-induced RV pressure overload in rats

The Ethics Committee of The General Hospital of Western Theater Command in Chengdu, Sichuan, China, approved all animal experiments in this study. In order to induce RV pressure overload, we elected to narrow the proximal pulmonary artery as previously described (17, 18). Twenty-two male SD rats were randomized to either sham operation (*n* = 7) or PAB (*n* = 15). 4-week-old male SD rats (100 g ± 9 g) were subjected to anesthesia utilizing a 2:1 volumetric ratio of isoflurane to air (7% induction, 3.5% maintenance), followed by endotracheal intubation and ventilation. Temgesic (buprenorphine) manufactured by Indivior UK Limited, Hull, UK, was administered subcutaneously to rats at a dose of 0.1 mg/kg. After shaving the chest region, open thoracic dissection was performed in the left upper quadrant to fully expose the RV outflow tract (RVOT). The carefully isolated pulmonary artery was then narrowed by proximal ligation with a fixed-size plastic ring (1.8 mm diameter). All animals underwent echocardiographic assessment of pulmonary artery pressure at 4 weeks after surgery to indirectly assess right heart function.

### Histological analyses

The cardiac specimens were meticulously fixed in a 4% paraformaldehyde solution to preserve tissue morphology and prevent enzymatic degradation. They were then embedded in paraffin to provide structural support for the sections. Subsequent histological analyses were conducted to assess the degree of cardiomyocyte hypertrophy [wheat germ agglutinin (WGA) staining] and fibrosis (Masson's trichrome staining), via employing a range of staining techniques.

### MiRNA sequencing analysis

Sample processing for RNA extraction involved the use of the miRNeasy Serum Kit (QIAGEN, Germany) for the extraction of high-quality RNA (including miRNAs, etc.) from serum samples, following the manufacturer's protocol. RNA integrity and concentration were subsequently assessed using an Agilent 2100 Bioanalyzer (Agilent, CA, USA). Library preparation is conducted with the MGIEasy Small RNA Library Prep Kit (BGI-Shenzhen, China). Prior to reverse transcription into cDNA and subsequent PCR amplification, RNA was sequentially ligated with 3’ and 5’ adaptors in two separate reaction systems. The library was first selected for fragment size using polyacrylamide gel electrophoresis, followed by quality control to ensure optimal conditions for sequencing. After this, single-stranded library products were generated through denaturation. A cyclization reaction was then performed to obtain single-stranded cyclized DNA products, while any remaining single-stranded linear DNA molecules were digested. The final single-stranded cyclized library underwent amplification using phi29 polymerase and rolling circle amplification (RCA), resulting in DNA nanoballs (DNBs) that contained over 300 copies of the original single-stranded cyclized library molecules. These DNBs were subsequently loaded into patterned nanoarrays, allowing for sequencing on the G400 platform (BGI, Shenzhen, China) to produce SE50 base length reads. This method ensures high sensitivity and accuracy for sequencing applications. After filtering the data to ensure we only have clean reads, we aligned these reads to the reference genome of the common rat (Rattus norvegicus) specifically version GCF_000001895.5_Rnor_6.0 from NCBI using Bowtie v2.2.5. Reads were compared with miRbase (using Bowtie2) and the Rfam database (using cmsearch) for non-coding RNA annotation. After miRNA expression data were normalized, the data were log2 transformed. Differential gene detection was conducted using DEseq2 with a Q-value threshold of ≤0.05, followed by the extraction and analysis of potential miRNA expression levels.

### RNA extraction, cDNA synthesis, and qRT-PCR

High-quality total RNA was carefully isolated from serum samples using TRIzol reagent (Invitrogen, Carlsbad, CA, USA) in strict compliance with the manufacturer's instructions. After extraction, we proceeded to synthesize complementary DNA (cDNA) from the total RNA. Specifically, in this step we utilized the Mir-X™ miRNA First-Strand Synthesis Kit (TaKaRa, Dalian, Liaoning, China) to reverse transcribe 0.5 μg of total RNA, which allowed us to obtain a more stable cDNA. To quantify the miRNAs present in our samples, we used the highly sensitive and reliable real-time PCR reagents TB Green® Premix Ex Taq™ II (TaKaRa, Dalian, Liaoning, China) with the Bio-Rad CFX Manager 2.0 software. To ensure the accuracy of the measurements, we normalized the transcript levels of miRNAs to the invariant internal reference gene U6 transcripts that were stably expressed across various conditions.

### Human study populations

Approval for the research was obtained from the Ethics Committee of The General Hospital of Western Theater Command in Chengdu, Sichuan, China. The ethical guidelines followed in this study were the World Health Organization Declaration of Helsinki. Inclusion criteria included most recent cardiac ultrasound data and pulmonary artery pressure or NYHA class III or IV symptoms based on the assessment of patient's primary cardiologist. Exclusion criteria included patients with a current or previous history of malignancy, pulmonary fibrosis, renal fibrosis, or inflammation condition. From March 2023 to June 2024, 40 patients with PH presenting with RV pressure overload (both adaptive and nonadaptive), 19 patients with LV hypertrophy, 20 patients with LHF, and 21 healthy controls were recruited from The General Hospital of Western Theater Command and The First Affiliated Hospital of Fujian Medical University in this study.

### Transthoracic echocardiography

Transthoracic echocardiography was performed to evaluate cardiac function, ventricular wall and septal thickness, and heart valve movement (19). Doppler and M-mode echocardiography were performed in rats using the Vevo 3100 LT ultra-high resolution small animal ultrasound imaging system (Fujifilm VisualSonics) with an MX250 transducer probe, and the rats were anesthetized using isoflurane (5% induction, 2% maintenance). Pulmonary artery blood flow acceleration time (PAT)/pulmonary ejection time (PET) was assessed by pulsed Doppler measurement. Right ventricular internal diameter (RVID) and diastolic right ventricular wall thickness (RVWT) and tricuspid annular plane systolic excursion (TAPSE) were measured by echocardiography.

### Right heart catheterization (RHC)

Rats were anesthetized with 2% pentobarbital sodium (40 mg/kg, intraperitoneal injection), and right ventricular systolic pressure (RVSP) was recorded by PowerLab system (AD Instruments) using a Millar catheter (Millar Instruments, US) inserted into the right ventricle through the right external jugular vein and the right atrium. The patient 's primary cardiologist performed RHC through the Swan-Ganz balloon tipped catheter (Edwards Lifesciences, Irvine, CA, USA) and collected data (20).

### Classification of the human study population

The study included 40 patients with RV remodeling in PH, 20 patients with LHF, and 19 patients with LV hypertrophy. The study cohort consisted of 34 male and 45 female patients whose medium age was 65 years [interquartile spacing (IQR) 59–70]. In addition, 21 serum samples were collected from healthy individuals with 12 females and 9 males with a median of 61 years of age (IQR 52–68). Statistical analysis showed that age and gender allocation did not differ considerably between the patient group and the healthy control group (*P* > 0.05). Blood samples were collected, immediately centrifuged and serum was stored at −80°C for later analysis. The criteria for adaptive RV remodeling include: TAPSE > 20 mm, Cardiac index(CI) >2.5 L/min/m^2^, Left ventricular ejection fraction (LVEF) >55%, Long-term RV afterload overload with PAPmean ≥ 25 mmHg, and the absence of left ventricular thickening i. e., interventricular septal thickness in diastole (IVSd) and left ventricular posterior wall thickness in diastole (LVPWd) both measuring less than 12 mm. The criteria for maladaptive RV remodeling include: TAPSE < 16 mm, CI < 2.2 L/min/m^2^, PAPmean ≥ 25 mmHg, RVEDd > 43 mm, LVEF > 55%, with both IVSd and LVPWd <12 mm. Inclusion in the LH hypertrophy group was conditional on the presence of chronic left ventricular afterload overload (which is usually caused by severe aortic stenosis). The screening criteria for this was: The mean aortic valve pressure gradient greater than 40 mmHg and/or aortic valve area less than 1.0 cm^2^, in addition to [IVSd] ≥12 mm, LVPWd ≥ 12 mm, TAPSE > 20 mm, LVEF > 55%, PAPmean < 25 mmHg, and RV end-diastolic basal diameter (RVD) <42 mm. The criteria for the LHF group include: LVEF < 50%, CI <2.2 L/min/m^2^, TAPSE > 20 mm, PAPmean < 25 mmHg, and RVD < 42 mm. The control group consists of healthy individuals who exhibit the absence of any alterations in either right and left ventricular function and structure.

### Statistical analysis

Continuous variables are represented as median values along with 25th–75th interquartile range (IQR). Categorical variables expressed as numbers and percentages. Comparisons between the two groups were conducted using the independent samples Student's *t*-test for normally distributed variables and the Mann–Whitney *U*-test for non-normally distributed variables. Multiple comparisons were analyzed by one-way analysis of variance (ANOVA) and non-normally distributed variables were tested using the Kruskal–Wallis test. Quantitative assessment of Masson's trichrome and WGA staining by Image J. ROC curves were plotted to evaluate the diagnostic accuracy of candidate differential miRNAs (dif-miRNA) for RV remodeling. The best miRNA-486 cut-off value for predicting maladaptive RV in PH patients was then calculated using the maximal Youden index (YI = Sensitivity + Specificity−1). Differences were determined statistically significant if a *P*-value or q-value of less than 0.05. Statistical analyses were conducted with the software SPSS 24.0.

## Results

### PAB successfully induced severe RV remodeling in rats

**Figure 1:**
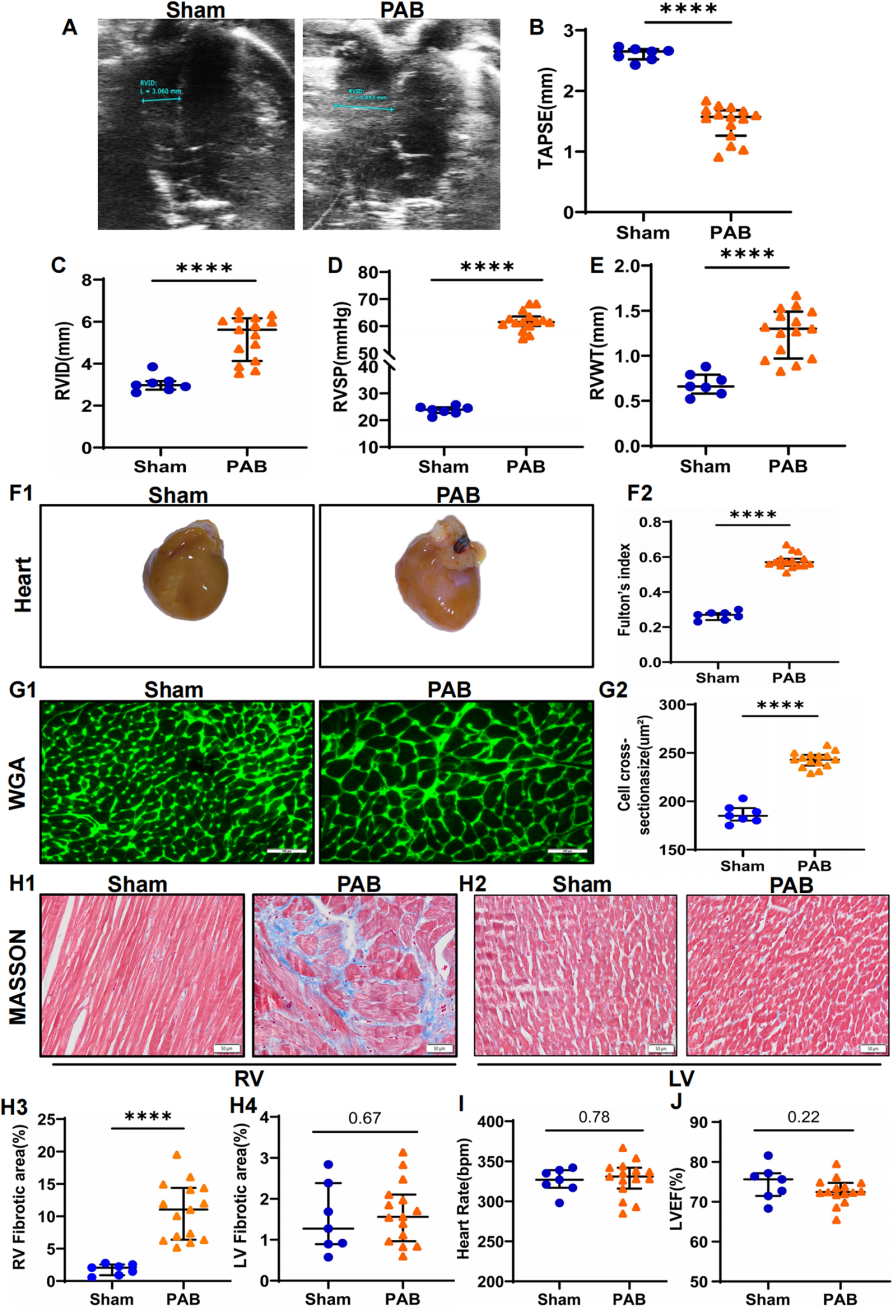
PAB leads to RV remodeling in rats. Rats were subjected to echocardiographic and histological analyses 4 weeks after sham or PAB surgery. **(A**–**C)** Quantification of echocardiographic analysis for right ventricular function. TAPSE: tricuspid annular plane systolic excursion; RVID: right ventricular internal diameter; ****p < 0.0001 (*n* = 7 for sham and *n* = 15 for PAB group). **(D)** Right heart catheterization to measure right ventricular pressure (RVSP) in rats; ****p < 0.0001 (*n* = 7 for sham and *n* = 15 for PAB group). **(E)** Ultrasonic measurements of RV free wall thickness (RVWT); ****p < 0.0001 (*n* = 7 for sham and *n* = 15 for PAB group). Representative gross heart images **(F1)** and the measurements of Fulton's index **(F2)**; ****p < 0.0001 (*n* = 7 for sham and *n* = 15 for PAB group). **(G)** Representative images of WGA staining images **(G1)** and quantification **(G2)** of cardiomyocyte cross-sectional area; ****p < 0.0001 (*n* = 7 for sham and *n* = 15 for PAB group). **(H)** Fibrosis of LV and RV measured by heart sections stained with Masson's trichrome staining **(H1-2)** and quantification of fibrotic area **(H3-4)**; ****p < 0.0001 (*n* = 7 for sham and *n* = 15 for PAB group). **(I**–**J)** Heart rate **(I)** and LVEF **(J)** in sham and PAB group (*n* = 7 for sham and *n* = 15 for PAB group).

The rat model of RV remodeling induced by PAB was established in this study. Compared to sham hearts, 4 weeks of PAB induced significant decrease in TAPSE and increase in RVID and RVSP (Figures 1A–D), indicating severe impairment in RV systolic function. Consistent with functional alterations, PAB induced obvious RV hypertrophic morphologic changes, as indicated by rises in diastolic RVWT, gross heart size, Fulton's index, and cardiomyocyte cross section area (Figures 1E–G). Masson's trichrome staining further showed pronounced RV fibrosis in PAB rats, but no significant fibrosis in the LV of rats (Figure 1H). There was also no significant difference in heart rate and left ventricular ejection fraction (LVEF) in the PAB group of rats compared to the sham group (Figures 1I–J**)**. These data suggest that PAB successfully induced RV remodeling in rats without resulting in LV functional and histological alterations.

### Discovery of novel miRNA biomarker candidates

**Figure 2:**
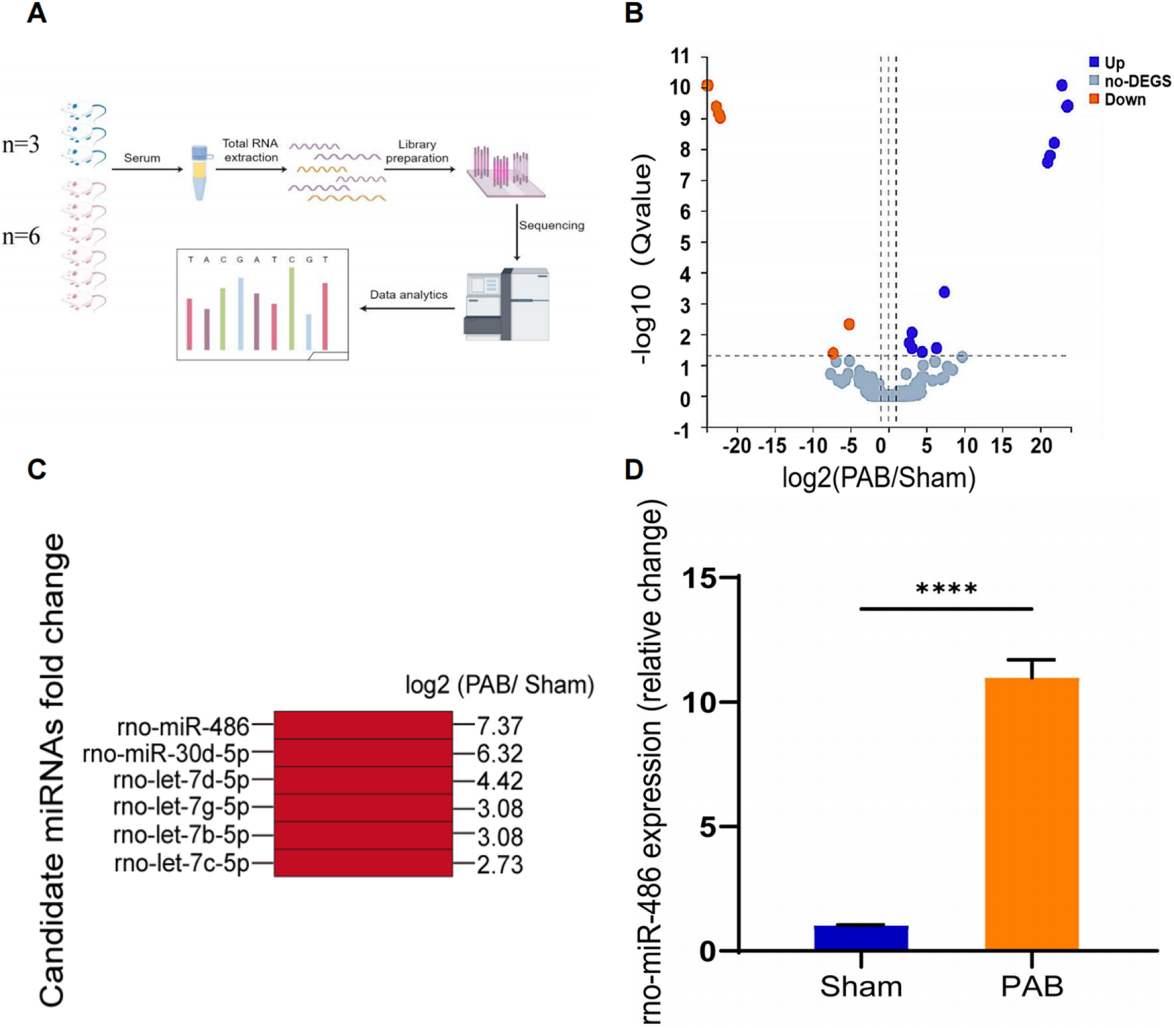
Profiling of serum miRNAs expression in RV remodeling via high-throughput sequencing and preliminary screening of candidate miRNAs. **(A)** Schematic diagram of the workflow of high-throughput miRNA sequencing (By Figdraw). **(B)** Volcano map showing comparison of serum miRNAs expression in sham- and PAB-operated rats. Differential analysis of genes was conducted using a differential analysis tool (DEseq2) and the Q-value and fold change (FC) for each gene was calculated (Qvalue ≤ 0.05, |log2FC|≥1) (up:12, down:7, no-DEGS:257). **(C)** MiRNA candidates and their corresponding fold changes. **(D)** The relative expression of miRNA-486 in rat serum 4 week after sham and PAB analyzed by RT-qPCR, ****p < 0.0001 (*n* = 6 for each group).

To screen potential serum miRNA biomarkers for RV remodeling, we performed high-throughput miRNA sequencing to profile dif-miRNAs. Differential analysis identified 19 dif-miRNAs in the sham-operated group (*n* = 3) and the PAB group (*n* = 6), with 12 upregulated and 7 downregulated, based on criteria of fold change (FC), log2FC ≥1, and q-value <0.05 (Figures 2A,B). Among the 12 upregulated dif-miRNAs, 6 miRNAs (i.e., rno-miR-122b, rno-miR-151-3p, rno-miR-215, novel-rno-miR352-3p, novel-rno-miR375-3p, novel-rno-miR425-3p) were excluded because of low overall sum of counts in individual samples. In the remain 6 upregulated dif-miRNAs, miRNA-486 exhibited highest upregulation (log2FC = 7.37, Figure 2C) and its great upregulation was further confirmed by RT-qPCR (Figure 2D). MiRNA-486 was chosen as the candidate biomarker for RV modeling, and its diagnostic efficacy was evaluated using clinical data from PH patients with RV remodeling, patients with LV hypertrophy, patients with LHF, and healthy individuals.

### Demographic and clinical characteristics of the study population

The characteristics of all subjects in this study and their subgroups (maladaptive RV, adaptive RV, LV hypertrophy, LHF and healthy controls) in terms of gender, age, BMI, cardiac ultrasonography measurements, and RHC measurements are shown in Table 1. The control group did not undergo invasive manipulation of RHC, therefore PAPmean and PASP were not measured. In patients with maladaptive RV, we observed significantly elevated levels of PASP, PAPmean and RVD values, as well as lower values for TAPSE, vs. individuals with adaptive RV, LV hypertrophy, LHF, and healthy controls. The adapted RV group exhibited significantly greater PASP and RVD relative to the LV hypertrophy (*p* < 0.0001 for both PASP and RVD), LHF (*p* < 0.0001 for both PASP and RVD) and the control group (*p* < 0.0001 for RVD). It should be noticed that differences in TAPSE values among the 4 groups were compared, and all of them had *p*-values > 0.05, which were not statistically significant.

**Table 1:** Clinical characteristics.

	All patients (*n* = 100)	Adaptive RV pressure overload (*n* = 20)	Maladaptive RV pressure overload (*n* = 20)	LV hypertrophy (*n* = 19)	LHF (*n* = 20)	Control group (*n* = 21)
Age, y, median (IQR)	65 (58–70)	65 (59–70)	68 (60–73)	65 (58–69)	66 (61–70)	61 (52–68)
Female sex, *n* (%)	57 (57)	11 (55)	11 (55)	11 (58)	12 (60)	12 (57)
BMI, kg/m2, median (IQR)	27 (23–29)	25 (22–30)	28 (24–32)	26 (22–29)	25 (23–28)	27 (25–30)
Diabetes, *n* (%)	34 (34)	5 (25)	6 (30)	9 (47)	11 (55)	3 (14)
NYHA ≥ III *n* (%)	36 (36)	4 (20)	7 (35)	11 (58)	11 (55)	3 (14)
CAD, *n* (%)	52 (52)	12 (60)	13 (65)	8 (42)	13 (65)	6 (29)
Right heart catheterization						
Cardiac index, L/min/m^2^, median (IQR)	2.3 (1.8–3.5)	3.3 (2.8–3.6)	1.8 (1.6–2.0)	2.2 (1.9–2.7)	1.8 (1.6–2.0)	3.8 (3.4–4.1)
PASP, mmHg, median (IQR)	48 (30–76)	67 (54–75)	87 (78–105)	31 (29–34)	30 (28–31)	n. a.
PAPmean, mmHg, median (IQR)	28 (18–47)	38 (34–46)	59 (49–65)	19 (16–23)	18 (15–20)	n. a.
Echocardiography						
LVEF, median (IQR)	62 (56–67)	65 (60–68)	62 (59–68)	65 (60–69)	39 (32–48)	63 (59–67)
IVSd, mm, median (IQR)	11 (10–13)	10 (9–11)	10 (9–11)	13 (12–14)	12 (11–13)	11 (9–12)
RVD, mm, median (IQR)	34 (28–44)	40 (34–44)	49 (47–51)	30 (26–37)	29 (25–35)	30 (28–33)
LVPWd, mm, median (IQR)	10 (9–12)	9 (8–10)	10 (9–11)	13 (12–14)	11 (10–12)	10 (9–11)
TAPSE, mm, median (IQR)	21 (20–24)	23 (21–26)	15 (12–16)	23 (21–25)	23 (21–25)	21 (20–23)
TAPSE/PASP, mm/mmHg, median (IQR)	0.54 (0.25–0.76)	0.36 (0.30–0.43)	0.16 (0.13–0.18)	0.70 (0.64–0.89)	0.77 (0.69–0.90)	n. a.
BNP, pg/ml, median (IQR)	324 (138–689)	315 (177–513)	897 (520–1,617)	146 (87–239)	724 (566–1,016)	103 (71–163)
miRNA-486, median (IQR)	1.65 (1.09–2.78)	2.46 (1.64–3.15)	4.46 (3.25–5.58)	1.20 (1.09–1.46)	1.79 (1.41–2.00)	0.95 (0.75–1.08)

BMI, body mass index; NYHA, New York Heart Association; CAD, coronary artery disease; PASP, pulmonary arterial systolic pressure; PAPmean, mean pulmonary artery pressure; LVEF, left ventricular ejection fraction; IVSd, diastolic interventricular septum thickness; RVD, right ventricular diameter; LVPWd, diastolic left ventricular posterior wall thickness; TAPSE, tricuspid annular plane systolic excursion.

### The relative expression of serum miRNA-486 and the concentration of BNP were significantly elevated in patients exhibiting maladaptive RV conditions

**Figure 3:**
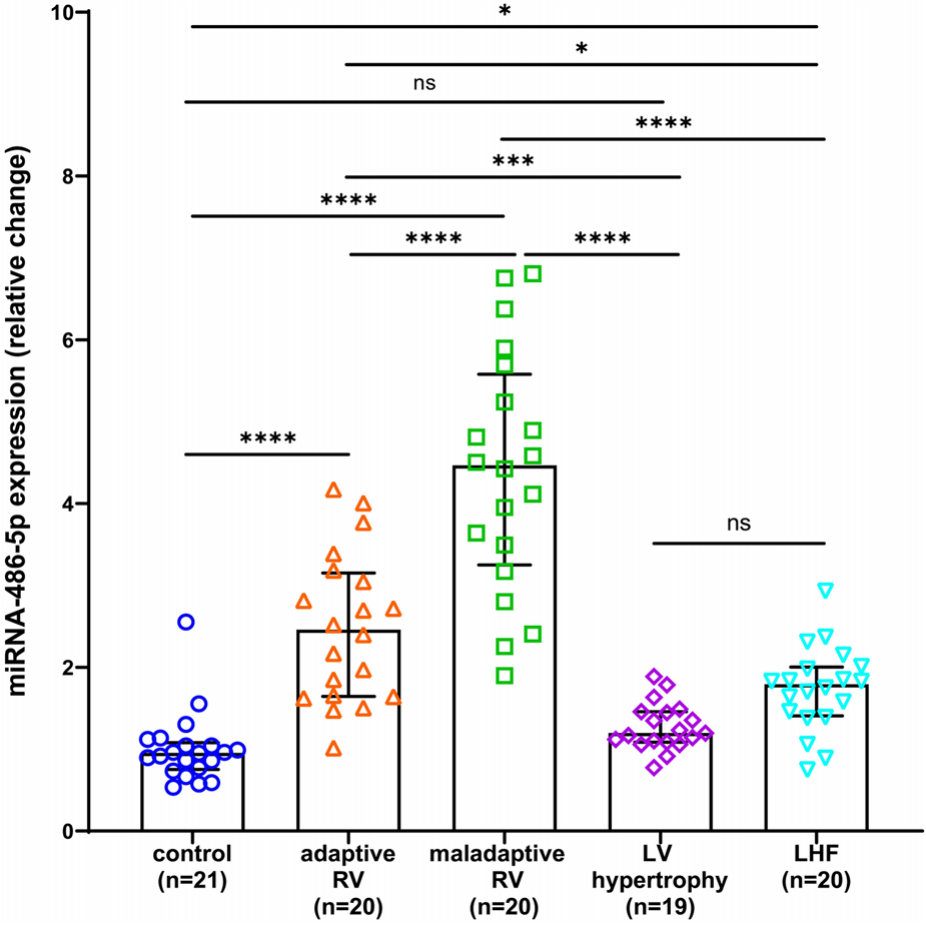
Box and scatter plots comparing of the relative expression of miRNA-486-5p in adaptive and nonadaptive RV, LV hypertrophy, LHF, and controls. ns, not significant, *p < 0.05, **p < 0.01, ***p < 0.001, ****p < 0.0001.

**Figure 4:**
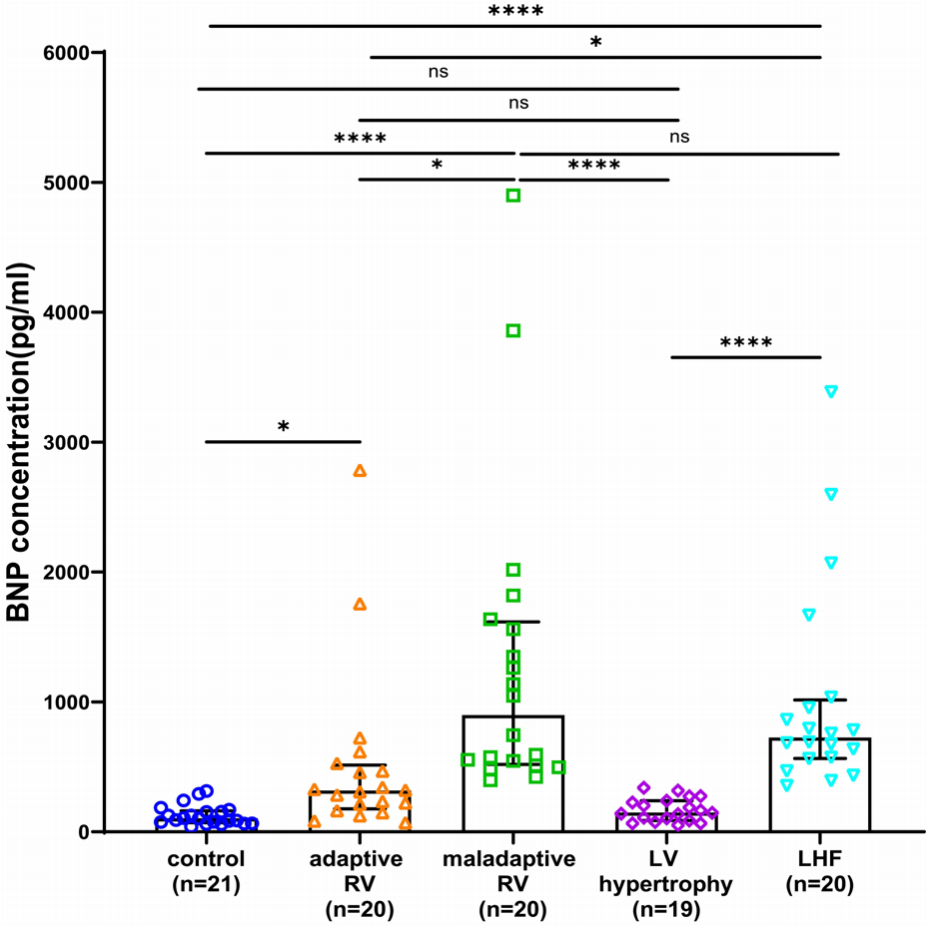
Box and scatter plots comparing of BNP concentrations in adaptive and maladaptive RV, LV hypertrophy, LHF, and controls. ns, not significant, *p < 0.05, **p < 0.01, ****p < 0.0001.

As miRNA-486 contains miRNA-486-3p and miRNA-486-5p in humans, we referenced the miRBase database and selected miRNA-486-5p for study because its sequence is consistent with ron-miRNA-486. Table 1 presents the median miRNA-486-5p levels and BNP concentrations for the total and each sub-population in this study, while Figures 3, 4 displays the results of statistical analysis for group comparisons. Relative to healthy controls, the serum miRNA-486-5p expression was remarkably enhanced in the adapted RV group (*p* < 0.0001), and further increased in those with maladaptive RV function (Figure 3). Serum miRNA-486-5p expression was mildly elevated in the LHF group compared with the control group but was still significantly lower than that in the adaptive RV group and the maladaptive RV group. It should be noticed that serum miRNA-486-5p level in LV hypertrophy group was not significantly different from control health (Figure 3).

As a contrast, BNP concentrations in the two RV groups and LHF group were significantly higher than control health, and the direct comparison revealed much higher BNP levels in the maladaptive group and LHF group compared with adaptive group (Figure 4). Notably, BNP was not significantly elevated in patients with LV hypertrophy compared with control health, and BNP was not significantly different in LHF compared with the maladaptive group (Figure 4).

### miRNA-486 as a diagnostic biomarker for maladaptive RV

**Figure 5:**
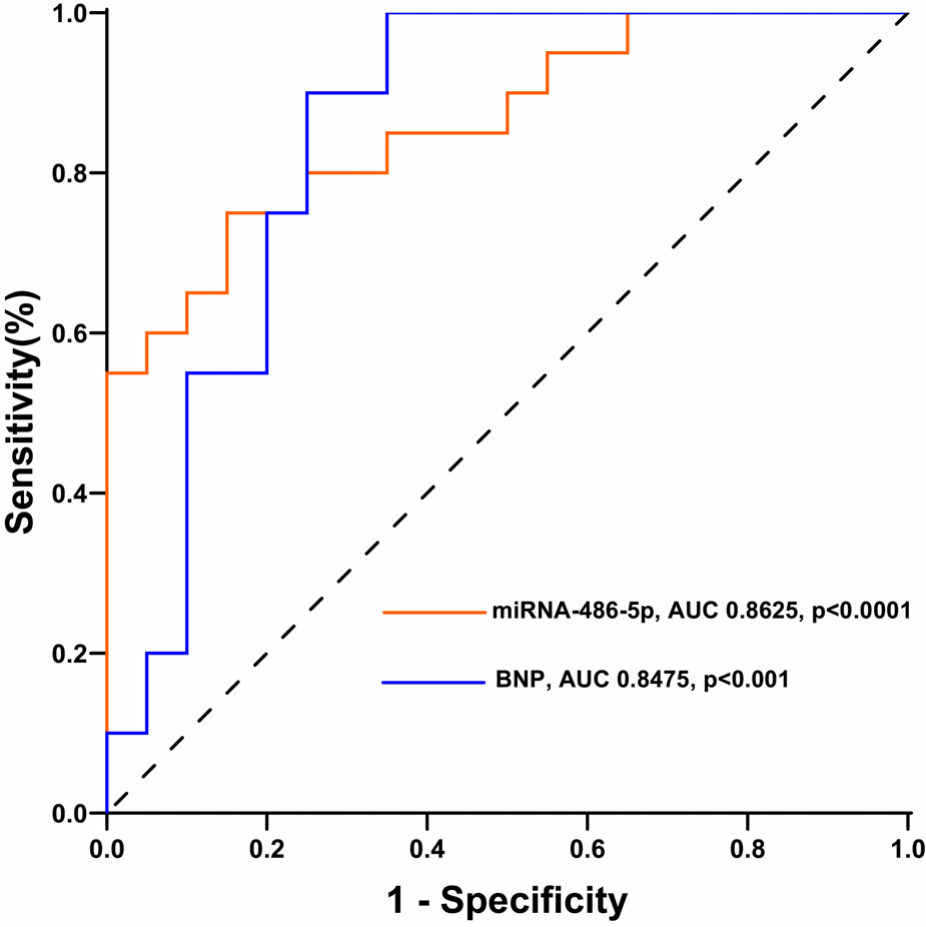
Receiver operating characteristics curves of miRNA-486-5p and BNP for maladaptive RV in PH patients.

**Figure 6:**
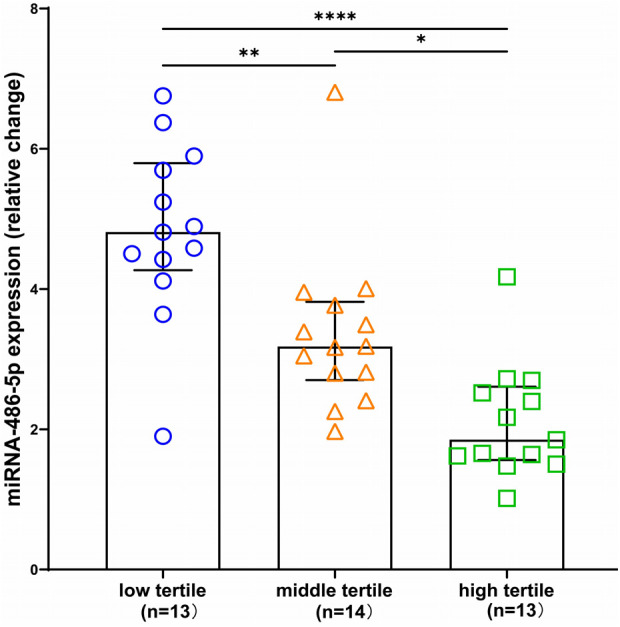
Box and scatter plots comparing of relative expression of miRNA-486 in low, medium and high TAPSE/PASP groups. *p < 0.05, **p < 0.01, ****p < 0.0001.

Next, we determined whether circulating miRNA-486-5p can be serve as diagnostic biomarker for maladaptive RV in PH patients. ROC curves were plotted and AUC values were calculated. As revealed by ROC analysis, miRNA-486-5p exhibited good diagnostic performance for maladaptive RV in PH patients, and the difference was not statistically significant when compared with BNP (Figure 5). The maximum Youden index (YI = Sensitivity + Specificity−1) was calculated from the ROC curve to obtain the optimal cut-off value of 3.441 for miRNA-486-5p. The patients with PH were further divided into low miRNA-486-5p (<3.441, *n* = 22) and high miRNA-486-5p group (≥3.441, *n* = 18) based on the optimal miRNA-486-5p cut-off value of 3.441. The clinical features of the two groups were displayed in Table 2. Elevated miRNA-486-5p levels correlated with reduced TAPSE and increased PASP, PAPmean, RVD, and BNP concentrations. Given that the TAPSE/PASP represents a novel metric for evaluating RV contractility and ventriculoarterial coupling in PH patients, we categorized PH patients into tertiles based on their TAPSE/PASP values: low (≤0.176 mm/mmHg), middle (0.176-0.318 mm/mmHg), and high (>0.318 mm/mmHg). Serum miRNA-486-5p levels were significantly lower in the high TAPSE/PASP tertile group with respect to both the low and middle TAPSE/PASP tertiles (Figure 6). Thus, these data suggest that circulating miRNA-486 could be a biomarker for diagnosing RV maladaptation in PH patients.

**Table 2:** Clinically relevant characteristics of patients (classified according to miRNA-486 truncation value).

	miRNA-486 < 3.441 *n* = 22	miRNA-486 ≥ 3.441 *n* = 18	*p*-value
Age, y, median (IQR)	66 (60–71)	66 (58–72)	0.91
Female sex, *n* (%)	12 (55)	10 (56)	0.95
BMI, kg/m^2^, median (IQR)	26 (22–31)	27 (24–31)	0.68
Diabetes, *n* (%)	5 (23)	5 (28)	0.72
NYHA ≥ III, *n* (%)	4 (18)	4 (22)	0.76
CAD, *n* (%)	13 (59)	12 (67)	0.63
Right heart catheterization			
Cardiac index, L/min/m^2^, median (IQR)	3.0 (2.4–3.5)	1.85 (1.70–2.15)	<0.001
PASP, mmHg, median (IQR)	67 (55–78)	85 (75–104)	<0.001
PAPmean, mmHg, median (IQR)	41 (36–49)	59 (47–65)	<0.001
Echocardiography			
LVEF, median (IQR)	64 (58–67)	66 (60–69)	0.28
IVSd, mm, median (IQR)	10 (9–11)	10 (9–11)	0.55
RVD, mm, median (IQR)	42 (37–47)	48 (47–50)	<0.001
LVPWd, mm, median (IQR)	9 (8–10)	10 (9–11)	0.49
TAPSE, mm, median (IQR)	22 (19–26)	15 (12–16)	<0.0001
TAPSE/PASP, mm/mmHg, median (IQR)	0.35 (0.26–0.42)	0.16 (0.13–0.20)	<0.0001
BNP, pg/ml, median (IQR)	398 (202–644)	821 (449–1,682)	0.0064
miRNA-486, median (IQR)	2.33 (1.65–2.80)	4.54 (3.99–5.74)	<0.0001

BMI, body mass index; NYHA, New York Heart Association; CAD, coronary artery disease; PASP, pulmonary arterial systolic pressure; PAPmean, mean pulmonary artery pressure; LVEF, left ventricular ejection fraction; IVSd, diastolic interventricular septum thickness; RVD, right ventricular diameter; LVPWd, diastolic left ventricular posterior wall thickness; TAPSE, tricuspid annular plane systolic excursion.

## Discussion

Given that deterioration of RV function is a critical determinant for mortality in PH, developing noninvasive diagnostic tools for maladaptive RV remodeling is crucial. MiRNAs, a category of highly conserved short non-coding RNAs, play a crucial role in regulating gene expression and are known to target above 60% of genes coding for human proteins (21). Contributions of MiRNAs in pathophysiological processes, disease diagnosis, pathogenesis, treatment and comment on prognosis is increasingly recognized due to their deregulated expression affecting gene profiles across various biological processes (22). Circulating miRNAs have been widely highlighted as promising diagnostic biomarkers due to their tissue specificity, relative stability in serum, and resistance to nuclease digestion and freeze-thaw cycles (23, 24). Numerous human microRNAs have attracted wide attentions because of their abnormal expressions in cardiovascular diseases. For example, Ovchinnikova ES and other colleagues found significant alterations in miR-18a, miR-223-3p and miR-652-3p in the serum samples of patients with acute heart failure (AHF) (25). However, it remains unclear whether MiRNAs can be used as biomarkers for the diagnosis of maladaptive RV remodeling.

Over the past few years, the efforts to identify noninvasive biomarkers for diagnosis of RV remodeling in PH were intensified (26–29). Our study is a part of the initiative focusing on circulating miRNAs due to their well-established association with cardiac remodeling. we are the first to report the profile of circulating miR-486 in the rat model of PAB-induced RV remodeling and in humans with adaptive and maladaptive PH, LV hypertrophy, LHF, and control health. The main findings of this study are: (1) Serum miRNA-486 levels were elevated in experimental models of RV pressure overload. (2) Serum miRNA-486 levels were higher in LHF patients than in controls but significantly lower than that in both RV groups; Serum miRNA-486 levels in patients with PH-induced maladaptive RV remodeling were higher than those in adaptive RV, LV hypertrophy, LHF and healthy controls, and there was no significant difference between LV hypertrophy and controls. (3) There is a close correlation between elevated serum miRNA-486 levels and RV dysfunction in patients with PH. Our data indicate that miRNA-486 may possess promise as a novel and specific biomarker for maladaptive RV remodeling in the context of PH. Although, the diagnostic performance of miRNA-486 was not better than BNP, our study provided another potential alternative option with lower cost.

miR-486 is a muscle-enriched miRNA with highly abundant in human serum samples (30–32). Previous studies have shown that miR-486-5p is highly expressed in myofibroblasts, cardiac muscle, and skeletal muscle, with moderately abundant in lung, brain, and bladder, and has been linked to a variety of diseases and organ fibrosis such as cancer and pulmonary fibrosis (30, 33–37). MiR-486-5p was found to have cardiac growth-promoting and muscle atrophy-preventing effects, which was achieved by targeting down-regulation of PTEN and FoxO1 (38). In addition, MiR-486-5p mitigates podocyte injury and renal fibrosis by targeting NFAT (39, 40). MiR-486-5p also plays an important role in inhibiting myocardial fibrosis and reducing excessive deposition of extracellular matrix proteins by specifically targeting and down-regulating the expression of Smad1, a key mediator of the transforming growth factor β signaling pathway (41). This suggests that miR-486-5p could be a potential therapeutic target for preventing or reversing fibrosis in heart disease. Based on these evidences, we speculated that elevated serum miRNA-486-5p in patients with PH-induced RV remodeling may in turn exert protective effect against RV remodeling, which needs to be elucidated in the future study.

Despite the promising results, our study also has several limitations: (1) The mild elevation of miRNA-486 in patients with LHF, to some extent, making its specificity as diagnostic marker for right heart remodeling limited to patients with PH. (2) A consensus on standardized internal controls for the measurement of serum miRNAs has not been established and validating candidate dif-miRNAs is more effectively achieved through absolute quantification. (3) This study only explored the value and significance of miRNA-486-5p as a biomarker for diagnosing maladaptive RV remodeling in PH patients, and further trials are needed to conduct more in-depth mechanistic studies and to explore whether they can suggest disease treatment efficacy.

## Data Availability

The original contributions presented in the study are publicly available. This data can be found here: https://www.ncbi.nlm.nih.gov/geo/query/acc.cgi, accession number: GSE287146.
